# Low-dose rituximab should be used for treating MS in resource-limited settings: Yes

**DOI:** 10.1177/13524585221089890

**Published:** 2022-04-18

**Authors:** Fredrik Piehl, Thomas Mathew

**Affiliations:** Department of Neurology, Karolinska University Hospital and Neuroimmunology Unit, Department of Clinical Neuroscience, Center for Molecular Medicine, Karolinska Institutet, Stockholm, Sweden; Department of Neurology, St. John’s Medical College Hospital, Bengaluru, India

Over the last two decades the treatment landscape for multiple sclerosis (MS) has evolved at an astonishing speed, now including more than a dozen unique disease-modifying therapies (DMTs) approved in the European Union/the United States.^1^ Different DMTs vary in efficacy, safety, tolerability, duration of effect and mode of administration, which provides greatly improved possibilities of tailoring treatment to individual needs, in turn improving long-term outcomes. In recent years, we have also witnessed a general shift towards the use of more highly effective DMTs, which reflects a growing awareness of the importance of early suppression of inflammatory disease activity to limit the risk of long-term disability accrual.^2^

Arguably, one of the greatest remaining challenges for the MS community is to increase access of treatment options also to patients who do not have public or private health insurance policies defraying drug and health care costs. In the US Medicaid programme, the cost of MS DMTs almost tripled from 2011 to 2017, reaching 1.32 billion USD, reflecting a shift to higher priced DMTs at the same time as availability of generic alternatives did not affect pricing significantly.^3^ Costs for MS DMTs increased more than for other neurological disorders, putting an increased financial burden on patients, where out-of-pocket costs might be more important than questions about effectiveness or side-effects.^4,5^ If this is a problem in rich world countries, what is the situation in less-affluent parts of the world? Well, first of all, we have much less information, as economic burden of MS mainly has been studied in high-income countries, where epidemiological and health economic data from low- and middle-income countries remains scarce.^6^ It is also evident that aside of a few exceptions such as HIV, a substantial change in pricing policies for drugs used to treat chronic conditions, such as MS, to improve access in resource-limited settings remains unrealistic. But there are other actions that lie within our reach, such as drug repurposing. Here the most interesting off-label alternative is rituximab, which is approved for rheumatoid arthritis and lymphoma, but also underwent formal clinical testing in both relapsing-remitting multiple sclerosis (RRMS) and primary progressive MS, for references see Ineichen et al.^7^ The market holder, however, chose to focus further development in MS on ocrelizumab, a highly similar biological with the same dosing schedule but commanding a much higher cost. Yet another biological, ofatumumab, used at low monthly subcutaneous doses, has now been approved. Collectively, there is now a wealth of data not only supporting the capacity of B-cell depletion to effectively suppress inflammatory disease activity in MS, but also in terms of safety.^7^

An unresolved issue, however, is what dosing regimen optimises long-term benefit–risk. Rituximab has been tested with bi-annual cycles of 1000 mg repeated after 2 weeks or approximately 500 mg repeated weekly over 4 weeks down to a small study with only a single infusion of 100 mg every 6 months, for references see previous works.^7,8^ It is noteworthy that rituximab has become the most used DMT in Sweden in spite of lacking a formal approval for this indication. Evidently this is not primarily due to economic factors, but instead was driven by early adoption of B-cell depletion as an attractive therapeutic option before approved alternatives became available. Thus, comparative observational studies across large real-world populations demonstrate that rituximab, mostly used at a dose of 500 mg every 6 months with no added benefit of higher doses, combines a high efficacy with acceptable safety and clearly superior tolerability compared with frequent MS approved DMTs.^7^ A further advantage with B-cell depletion compared with, in particular, cell migration modulators is that termination of treatment is not associated with rebound phenomena,^9^ which may be of additional importance in resource-limited contexts. We should also proud ourselves that the academic community has taken up the challenge to provide more formal proof of the comparative benefit of rituximab versus other DMT options. The RIFUND trial (clinicaltrials.gov identifier: NCT02746744), due to be reported soon, compares the effect of rituximab 500 mg every 6 months with dimethyl fumarate, while the RIDOSE study (NCT03979456) compares effect and safety with a 500 mg rituximab dose every 6 months with yearly infusions. Additional examples include Norwegian and Danish initiatives comparing rituximab 500 mg (OVERLORD; NCT04578639) or 1000 mg (DanNORMS; NCT04688788) every 6 months with ocrelizumab 600 mg, respectively. With these efforts the evidence base for rituximab in RRMS is set to increase further and, notably, also include highly effective comparators. Should these results be awaited before including low-dose rituximab among possible treatment alternatives in resource-limited settings? We argue this should not be the case. It is futile to dispute that rituximab is less effective in RRMS than regular platform therapies, which are the most affordable among MS-approved DMTs. Furthermore, the use of a rituximab biosimilar at low dose, especially with an infrequent dosing schedule, is substantially cheaper than platform alternatives. Importantly, there is already existing evidence that use of rituximab appears effective, safe and affordable in the treatment of MS in resource-limited settings.^10^ On the contrary, newly approved B-cell therapies, such as ocrelizumab and ofatumumab, are not easily accessible, and the prohibitive costs of these medications will anyway prevent their usage by most MS specialists in the low- and middle-income countries. Therefore, the high efficacy, long duration of action, infrequent injections, patient acceptability, favourable safety profile, availability of low-cost biosimilars, and a long experience all favours the use of rituximab in the treatment of MS, especially in resource-limited settings.^10^

In conclusion, to withhold the option of rituximab for an MS patient in the developing world, where the patient him/herself must cover most or all of the financial burden, is to exert double standards if it constitutes a relevant therapeutic alternative for a patient in the Nordics and certain other rich world locations. Within the MS community, this could be our contribution towards fulfilling the United Nations 2030 Sustainable Development Goals, for example, good health and reduced inequality.
